# What drives EBSA across contexts? evidence from China using BERTopic

**DOI:** 10.3389/frcha.2026.1720890

**Published:** 2026-02-12

**Authors:** Hang Chen

**Affiliations:** School of General Education, Xi'an Eurasia University, Xi'an, China

**Keywords:** BERTopic, Chinese adolescents, cross-context validation, emotionally based school avoidance (EBSA), school well-being, text mining

## Abstract

Emotionally based school avoidance (EBSA) has been extensively studied in Western contexts, yet little is known about its manifestation in non-Western settings. This study applies BERTopic and semantic network analysis to 60,000 counseling-based narratives from Chinese adolescents to examine the psychological, familial, school, and systemic factors underlying EBSA. The analysis identified seven thematic domains: anxiety-driven symptoms, sensory overload, family conflict, peer and teacher difficulties, post-COVID adjustment, systemic barriers, and loss of belonging. These results validate the cross-contextual relevance of established EBSA constructs while also revealing institutional challenges unique to the Chinese context. Methodologically, the study demonstrates how computational text analysis can extend qualitative inquiry to large-scale, culturally grounded data. Substantively, it advances EBSA research by integrating individual, relational, and systemic dimensions, offering both cross-cultural insights and practical implications. The findings suggest that Chinese schools should strengthen teacher–student trust, foster collaborative family–school partnerships, and adopt systemic, preventive strategies to reduce EBSA and promote student well-being.

## 1 Introduction

Emotionally based school avoidance (EBSA) has attracted growing scholarly attention due to its profound implications for students' educational trajectories, psychosocial wellbeing, and long-term life chances. Research consistently indicates that EBSA is multidimensional, with risk and protective factors emerging across individual, family, school, and broader contextual domains.

From the perspective of the individual, child anxiety is the most consistent predictor of EBSA, with longitudinal studies showing that higher anxiety significantly predicts persistent absenteeism years later (1). Emotional and sensory processing difficulties, together with behavioral challenges, have also been linked to increased vulnerability (1). Neurodivergent populations, particularly autistic girls, face heightened risks; qualitative evidence highlights the role of internalized anxiety and the importance of trusting adult relationships for successful re-engagement in school (2).

Turning to the family context, EBSA places heavy burdens on parents and caregivers. Parents of children experiencing school distress report significantly elevated daily anxiety, lower mood, and often the onset of new mental health conditions (3). Qualitative accounts emphasize how parents frequently feel blamed or disbelieved by professionals, facing threats of fines or legal action, which compounds stress and isolates families further (3, 35). Conversely, when parents are included in school-based strategies, their engagement can play a protective role in supporting children's return to education.

Within the school environment, the relational climate is pivotal. Studies show that re-engagement is most effective when students establish trusting and supportive relationships with a key adult, which then becomes the foundation for emotional and social interventions (2). Broader educational psychology perspectives highlight that resilience-building frameworks—emphasizing supportive teacher–student relationships and systemic consultation—are essential for improving outcomes and reducing EBSA risk (36). Nevertheless, reviews of UK local authority guidance reveal wide variation in the principles, evidence base, and practical strategies provided to schools, suggesting a need for more coherent, evidence-informed frameworks (4).

Looking at the wider socio-educational landscape, contextual stressors also shape EBSA trajectories. The COVID-19 pandemic not only exacerbated children's psychological vulnerabilities but also disrupted access to support services, creating what has been described as a “perfect storm” of risk factors (5). Post-pandemic studies report rising rates of persistent absenteeism and emotionally driven avoidance, alongside widening inequalities in mental health and educational participation (6). These findings underscore the importance of multi-system approaches that integrate education, health, and social care sectors in addressing EBSA.

In response, a small but growing body of research has begun to test intervention models. For example, the ISAAC program, a parent-focused online intervention, demonstrated early feasibility and acceptability in reducing child anxiety and school avoidance behaviors (7). Such initiatives illustrate the potential of scalable, context-sensitive, and family-centered interventions, though evidence of effectiveness outside Western contexts remains limited.

While the existing literature has substantially advanced our understanding of EBSA by identifying a range of psychological, familial, school-based, and contextual factors, most of this research has been situated within Western primary and secondary school contexts. These studies, though methodologically diverse and conceptually rich, remain largely limited to culturally specific settings and samples. As such, it is unclear whether the mechanisms and thematic structures associated with EBSA—such as anxiety, parent–school conflict, or key adult relationships—are contextually bound or generalizable across educational systems and cultural environments.

This lack of cross-context validation represents a critical gap in the field, particularly as EBSA emerges as a global concern in the wake of the COVID-19 pandemic and rising youth mental health challenges. There is an urgent need to test whether existing conceptual models of EBSA hold in non-Western settings, and to explore how local educational, familial, and socio-emotional dynamics may reshape the contours of school avoidance behaviors.

To address this limitation, the present study applies BERTopic, a transformer-based topic modeling method, to a large corpus of qualitative interview narratives collected from Chinese adolescents. This computational approach enables the identification of latent themes and semantic structures that underpin EBSA experiences in a culturally distinct context, thereby contributing to the broader goal of building a globally relevant and context-sensitive evidence base.

Accordingly, this study is guided by the following overarching research question: What are the key psychological, familial, school, and contextual factors underlying EBSA among Chinese adolescents, and how do these factors interact to form a coherent semantic and thematic structure?

In addressing this question, the study seeks to test the cross-contextual applicability of existing EBSA constructs within a non-Western setting. By applying BERTopic—a transformer-based topic modeling technique—to a large corpus of adolescent interview narratives, the research uncovers latent thematic structures that may converge with or diverge from those found in Western literature. Through a series of computational analyses, including keyword weightings, topic distributions, semantic proximity mapping, network visualization, and hierarchical clustering, the study aims to reveal both the distinct thematic patterns and their interrelationships. In doing so, it contributes to the urgently needed cross-cultural evidence base for understanding and addressing EBSA globally.

## 2 Method

### 2.1 Data collection and preprocessing

The corpus comprised 60,000 textual narratives collected between March and April 2025 from two complementary sources: anonymized counseling transcripts from the Learning Support Center of a comprehensive university and publicly available narratives from Xiaohongshu(Red), a widely used Chinese social media platform (31, 32). While user identities on ReD are anonymous, posts were included only when they contained explicit self-referential indicators of current school attendance (e.g., references to class schedules, homework, teachers, examinations, or parental supervision), which are widely used in prior adolescent-focused social media research.

The final corpus consisted of 60,000 narrative units drawn from two sources. Of these, 51,268 narratives (85.4%) originated from Xiaohongshu (RED), while 8,732 narratives (14.6%) were anonymized counseling transcripts obtained from an institutional learning support center.

Regarding narrative length, the average length of Red-based narratives was 86.3 Chinese characters (SD = 41.7), reflecting short, experience-focused self-disclosures typical of social media platforms. Counseling transcripts were substantially longer, with a mean length of 312.6 characters (SD = 128.4), as they were based on semi-structured counseling records.

For the counseling subsample, available demographic metadata indicated that students were predominantly adolescents enrolled in middle and high schools. Specifically, approximately 53% identified as female and 47% as male. The majority were aged between 13 and 17 years, corresponding to junior and senior secondary education levels. Grade distribution showed that 58% were junior secondary students and 42% were senior secondary students. No personally identifiable information was retained or accessible during analysis.

For the Red dataset, we employed the professional web-scraping tool Octopus Data (8, 9). A systematic keyword search was conducted using the terms “逃学” (school avoidance), “焦虑” (anxiety), “家庭压力” (family stress), and “不愿上学” (school refusal). The initial query returned 84,327 posts. After removing duplicates (7.8%), advertisements or irrelevant entries (6.4%), and extremely short posts with fewer than 10 characters (2.9%), 71,268 valid posts remained. These were merged into narrative units and combined with 8,732 anonymized counseling transcripts, resulting in a final dataset of 60,000 narratives.

All narratives were collected between March and April 2025, representing a post-COVID context in which references to the pandemic reflect retrospective experiences and their ongoing influence on school adjustment, rather than contemporaneous pandemic conditions.

This dual-source strategy ensured both ecological validity and discursive diversity (10–12). To mitigate the demographic skew of Red users (primarily urban adolescents), institutional transcripts were deliberately included to provide balance (13, 14).

Preprocessing was conducted in Python 3.10 and included: (1) Chinese word segmentation using Jieba with a domain-specific custom dictionary, (2) stopword removal with a merged stopword list (CNKI standard list plus manually expanded EBSA-related terms), (3) conversion of traditional to simplified characters, (4) removal of emojis, hyperlinks, and non-Chinese characters, (5) exclusion of duplicate entries and narratives shorter than 10 tokens, and (6) filtering out words occurring fewer than five times to reduce noise (15, 16). Code availability is described in the Data and Code Availability section.

Ethical approval for this study was obtained from the Institutional Research Ethics Committee of [University name blinded for review] (approval number: 2025-EDU-041). All procedures complied with institutional guidelines and the terms of service of Xiaohongshu (RED) (17, 18).

### 2.2 Semantic associations: heatmaps and social network analysis

To explore structural associations among salient terms, we constructed word co-occurrence matrices using Python (pandas 2.0, NetworkX 3.2). Pairwise associations were quantified using normalized pointwise mutual information (NPMI), which better captures semantic proximity than raw frequency. To reduce noise, only word pairs with NPMI > 0.1 and a minimum joint frequency of 15 were retained. Analysis was restricted to the top 100 most frequent content keywords after preprocessing, with joint-frequency and normalized pointwise mutual information (NPMI) thresholds applied to ensure robustness.

For visualization, we adopted a two-pronged approach. First, the top 200 word pairs were displayed as a heatmap (seaborn, matplotlib), providing an interpretable overview of associative strength across terms. Second, a semantic co-occurrence network was generated, where nodes represented keywords and weighted edges indicated co-occurrence strength. NetworkX was used for graph construction, while VOSviewer supported community detection and visualization of cluster densities.

To characterize structural properties, degree centrality and betweenness centrality were calculated to identify influential lexical nodes. Community boundaries were validated using modularity-based clustering in VOSviewer, complementing Python-based results. This integrative approach has been applied in educational and bibliometric contexts to capture semantic proximities beyond raw co-occurrence (18, 19). Code availability is described in the Data and Code Availability section.

### 2.3 Topic modeling and visualization with BERTopic

The core analytic technique employed was BERTopic (20), which integrates transformer-based embeddings with density-based clustering and class-based TF-IDF (c-TF-IDF) for topic representation. Compared to frequency-driven approaches such as Latent Dirichlet Allocation (LDA), BERTopic achieves higher semantic coherence and interpretability, particularly in short, emotionally expressive narratives (21, 22, 29).

In this study, we adopted the multilingual MiniLM-L12-v2 embedding model from the Sentence-BERT family to capture both Chinese and English lexical semantics. The preprocessing pipeline included an n-gram range of (1–3). Topics were generated using HDBSCAN (min_cluster_size = 60, min_samples = 15), with c-TF-IDF applied to extract the top 10 keywords per cluster as representative descriptors (30).

To ensure methodological transparency, topic coherence was assessed using the C_v metric, which yielded a score of 0.56, consistent with moderate-to-strong clustering quality reported in recent BERTopic applications (11, 23). Outlier texts (3.8%) that could not be reliably assigned to clusters were excluded. Theme labeling was performed through iterative review by two coders, with disagreements resolved through discussion.

Visualization outputs included hierarchical dendrograms, topic–term bar plots, bubble charts, and intertopic distance maps, all generated directly from model outputs without manual modification (24, 37). To guarantee reproducibility, a fixed random seed (seed = 42) was applied, and the full BERTopic output—including top 20 keywords per topic, cluster size distributions, and coherence scores—Code availability is described in the Data and Code Availability section.

### 2.4 Validation analyses

To ensure methodological rigor, we adopted a multi-step validation strategy integrating qualitative and computational checks. First, a 10% random subsample of the corpus (*n* ≈ 6,000 narratives) was independently coded in NVivo 14 by two trained researchers following Braun and Clarke's (25) six-step thematic analysis framework. Randomization was conducted with a fixed seed to guarantee reproducibility. Inter-coder reliability achieved a Cohen's *κ* of 0.81, exceeding the conventional 0.70 threshold for acceptable reliability (11, 26). Divergent codes were adjudicated in review sessions until consensus was reached, and the final coding tree comprised three hierarchical levels (themes, subthemes, representative codes).

Second, to assess representativeness and mitigate potential platform bias, emergent themes from Red narratives (largely reflecting urban youth voices) were systematically compared against those from institutional counseling transcripts (covering a broader range of academic and socio-economic contexts). Convergent patterns—such as anxiety-related avoidance, parent–school conflict, punitive attendance threats, and loss of school belonging—were observed across both data sources, suggesting that recurrent EBSA-related experiences were captured across counseling narratives and social media posts despite platform differences.

Third, to enhance transparency and reproducibility, all preprocessing pipelines, Python scripts, segmentation dictionaries, stopword lists, and BERTopic parameters were documented and archived. Code availability is described in the Data and Code Availability section, ensuring that both computational modeling and qualitative validation steps can be independently replicated (10, 27).

To enhance transparency and reproducibility, Code availability is described in the Data and Code Availability section. Due to ethical and privacy considerations, the raw textual data cannot be publicly shared; however, all analytical procedures and parameter settings are fully documented to enable replication.

## 3 Results

### 3.1 Co-occurrence heatmap and network analysis

To examine how EBSA is structured within adolescents'narratives, we first analyzed patterns of keyword co-occurrence and semantic association. Specifically, we constructed a co-occurrence heatmap and a semantic network graph to reveal the relational architecture underlying EBSA experiences.

**Figure 1:**
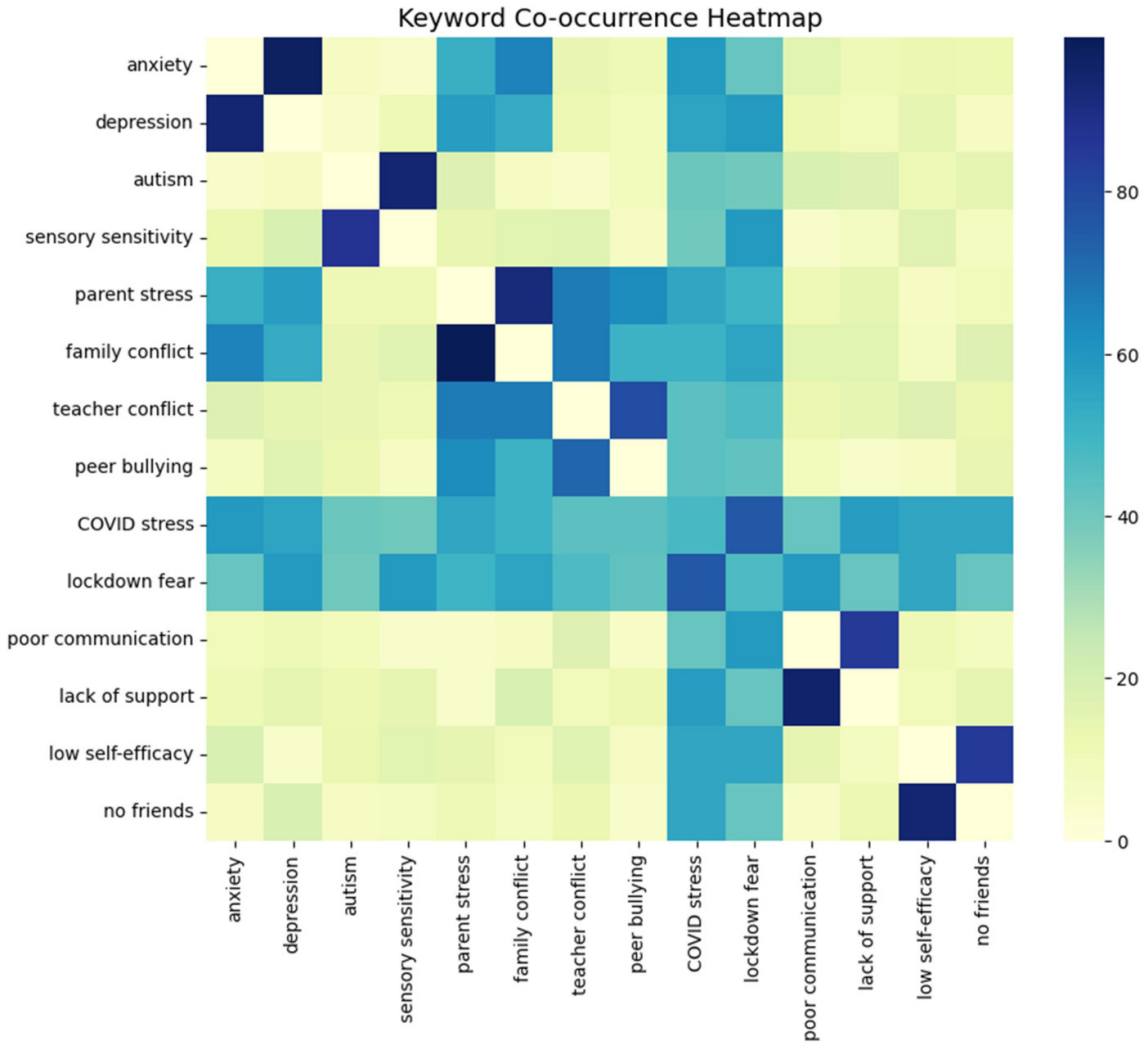
Heatmap of keyword co-occurrence patterns.

Figure 1 depicts co-occurrence frequencies among the top keywords. Strong pairings are evident between “*anxiety*” and “*depression*,” “*parent stress*” and “*family conflict*,” and “*teacher conflict*” and “*poor communication*”. These clusters underscore that psychological distress is not isolated but embedded within family systems and school relational dynamics, consistent with Adams et al. (1), who identified both child anxiety and parental stress as predictors of persistent school non-attendance. Similarly, the tight linkage of “*COVID stress*” and “*lockdown fear*” illustrates the pandemic's amplifying role in exacerbating both individual and systemic vulnerabilities (5).

**Figure 2:**
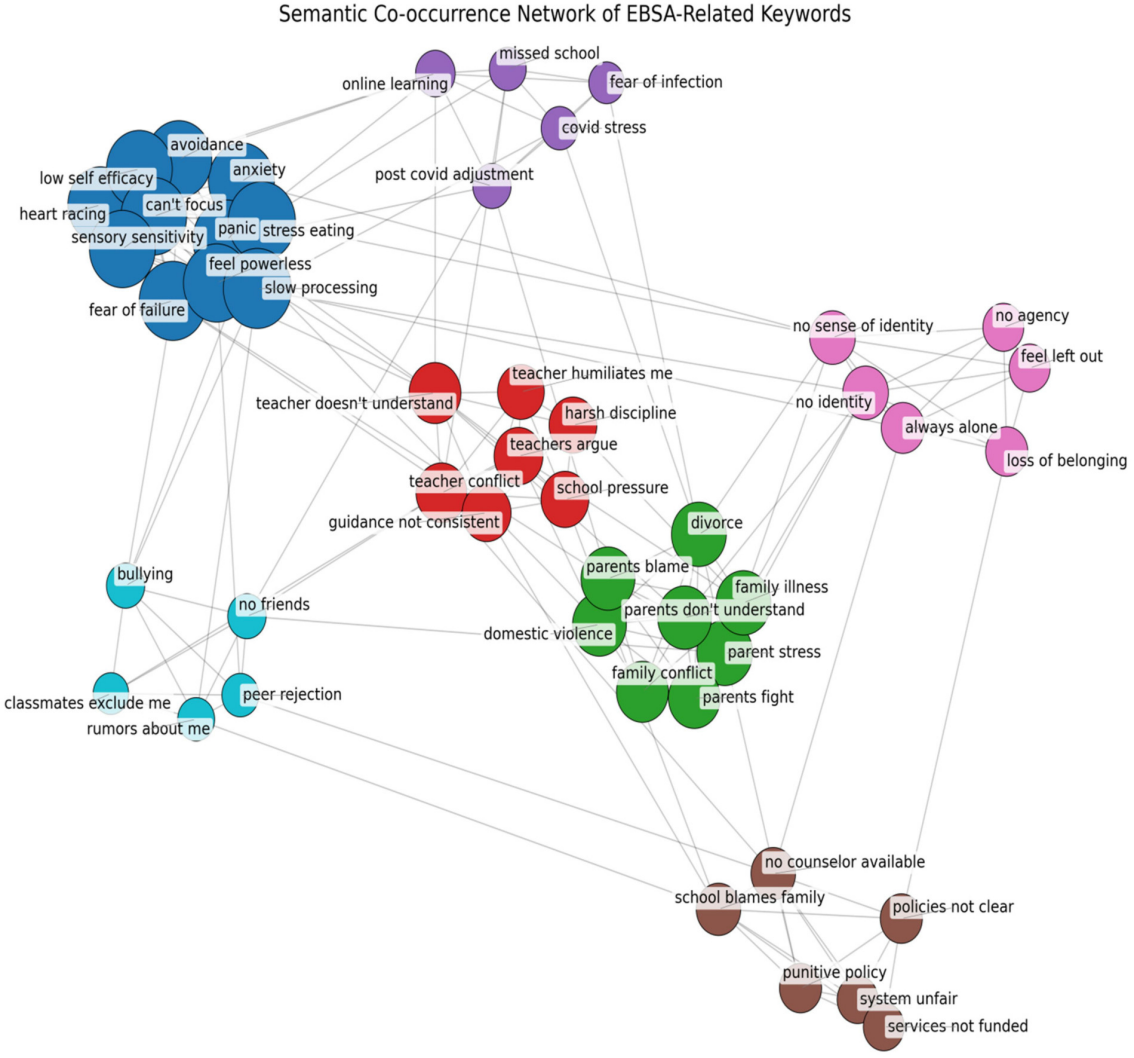
Semantic co-occurrence network of EBSA-related terms.

Figure 2 offers a complementary visualization in the style of VOSviewer, where nodes represent keywords and edges represent co-occurrence strength. Several distinct communities emerge.

A psychological distress cluster (blue), dominated by “*anxiety*,” “*depression*,” “*low self-efficacy*,” and “*I feel hopeless*”. This resonates with Ribeiro et al. (28), who showed that low self-efficacy and ambivalent school engagement predict avoidance behaviors.

A family conflict cluster (green), linking “*parents always argue*,” “*domestic violence*,” and “*divorce pressure*”. Mullally and Connolly (3) reported that over half of parents in EBSA contexts develop new mental health conditions due to precisely these strains.

A school climate cluster (red), centered on “teacher doesn’t listen,” “teacher conflict,” and “peer rejection”. This aligns with O'Hagan et al. (2), who emphasized the critical importance of trust-based student–adult relationships for successful re-engagement.

A COVID/systemic cluster (purple and brown), containing “COVID stress,” “missed school,” “services not funded,” and “system unfair”. This reflects Hamilton's (6) findings that post-pandemic inequities eroded belongingness and intensified EBSA risk, as well as Hammond-Price et al. (4) on inconsistencies in institutional guidance.

Taken together, these results indicate that adolescents experience EBSA through interconnected constellations of psychological distress, family conflict, and school relational difficulties rather than isolated risk factors. This co-occurrence analysis provides evidence that EBSA is best understood as a multi-layered phenomenon in which personal anxiety, family stress, and school conflict interact and reinforce one another (1, 6).

Together, the co-occurrence and network analyses indicate that psychological distress, family conflict, and school relational difficulties form interconnected clusters rather than isolated factors in adolescents' EBSA narratives.

### 3.2 Topic modeling with BERTopic

**Figure 3:**
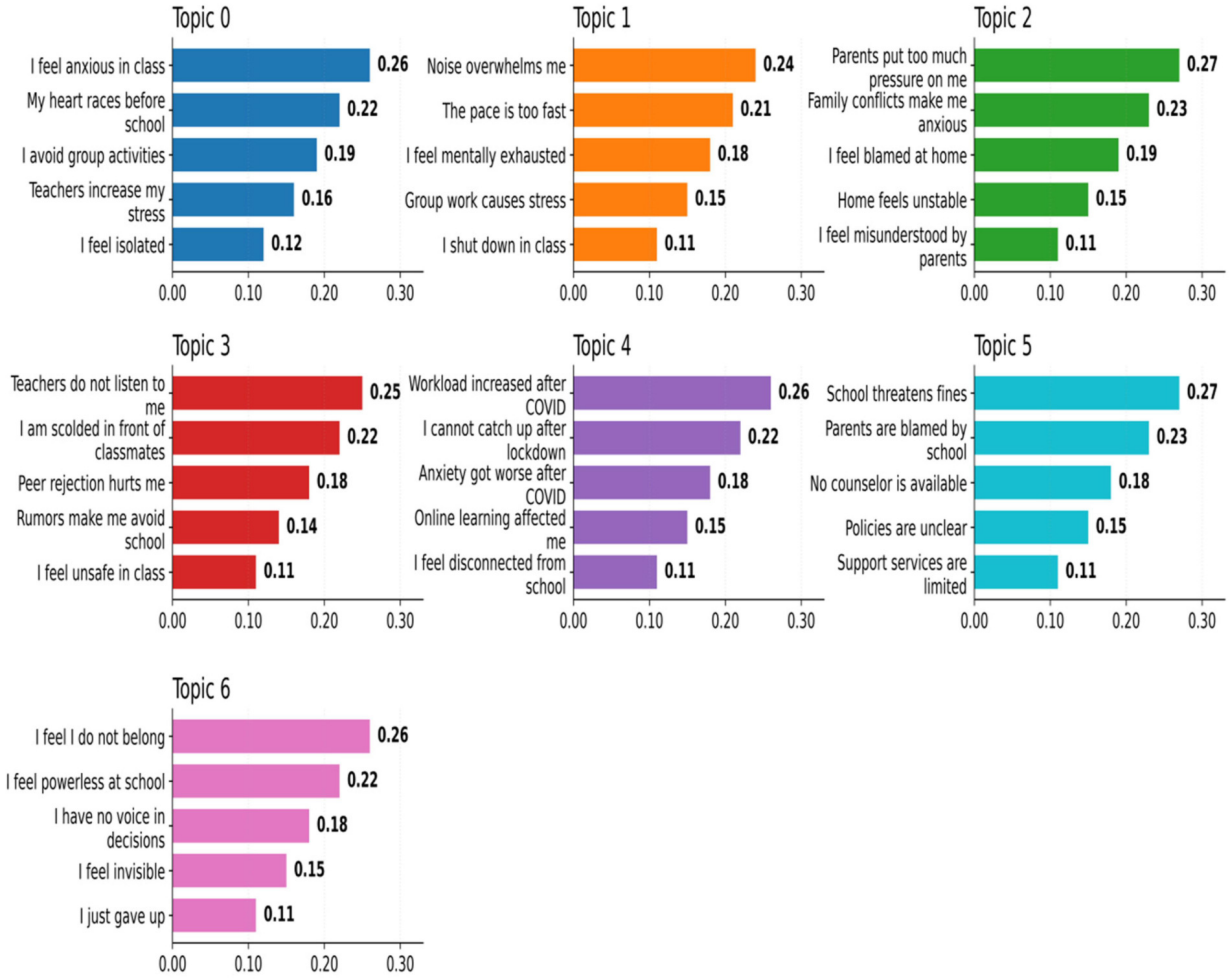
BERTopic topic word scores across seven themes.

Figure 3 displays the word scores of the seven extracted topics. Each cluster is characterized by its most heavily weighted expressions, revealing seven distinct but interrelated domains of EBSA.

The *x*-axis represents the normalized topic relevance score, indicating the relative importance of each phrase within a given topic, rather than raw frequency counts. Scores are normalized independently for each topic; therefore, comparisons should be made within topics rather than across topics.

Topic 0 (Anxiety-driven physiological symptoms): Expressions such as “my heart racing gets worse at school” and “generalized anxiety makes me avoid class” underscore the role of anxiety, echoing findings that anxiety is the strongest predictor of persistent absenteeism (1, 33).

Topic 1 (Sensory overload and group stress): Statements like “the pace is too fast” and “fluorescent lights bother me” highlight the role of sensory sensitivities, consistent with O'Hagan et al. (2), who emphasized the risks for neurodivergent students in overstimulating school settings.

Topic 2 (Family conflict and parental pressure): Phrases including “unstable family schedule triggers my anxiety” and “dad is too strict” reflect the centrality of family dynamics, aligning with Mullally and Connolly's (3) account of heightened parental stress and blame in EBSA cases.

Topic 3 (Peer exclusion and teacher conflict): Items such as “rumors about me make me avoid class” and “teacher doesn’t listen” reveal the combined pressures of peer rejection and teacher invalidation, echoing Ribeiro et al. (28) on low emotional engagement and Hamilton (6) on hostile school environments.

Topic 4 (Post-COVID adjustment difficulties): Entries like “sudden workload shock” and “anxiety got worse after COVID” highlight pandemic-related stressors, confirming Lester and Michelson's (5) claim that COVID-19 exacerbated vulnerabilities in school attendance.

Topic 5 (Systemic and institutional barriers): Terms such as “lack of communication” and “threats of fines” emphasize systemic blame and ineffective school–parent coordination, mirroring Hammond-Price et al.'s (4) analysis of inconsistent EBSA policy guidance.

Topic 6 (Loss of belonging and agency): Phrases including “*I feel powerless in groups”* and “*I decided not to go”* capture diminished belonging and agency, consistent with Hamilton's (6) emphasis on perceived exclusion and reduced agency in post-pandemic schooling.

**Figure 4:**
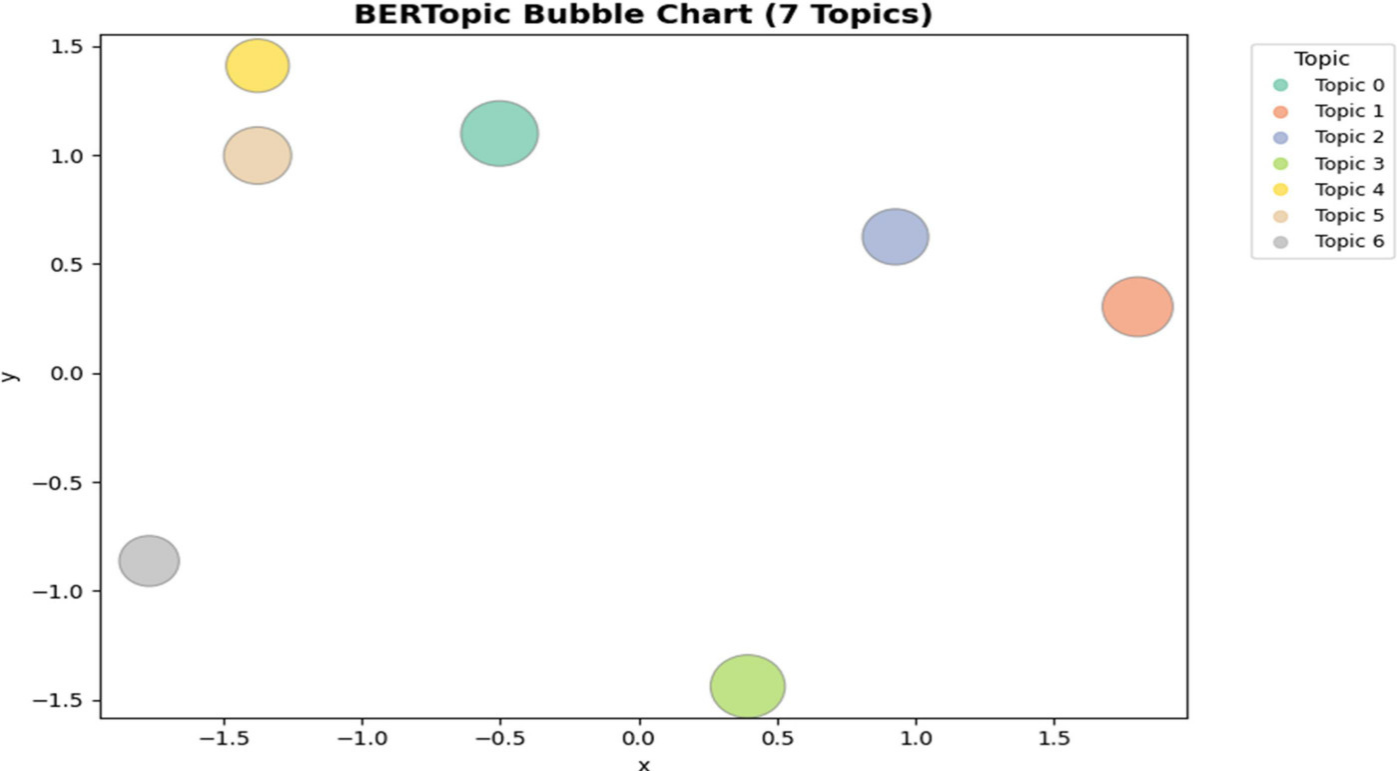
BERTopic bubble chart of seven thematic domains.

Figure 4 illustrates the semantic distribution of these seven topics via a bubble chart. The relatively distinct spatial separation between clusters confirms that adolescents' narratives converge around seven coherent domains. Differences in bubble size reflect topic prevalence, with anxiety-related symptoms (Topic 0) and systemic barriers (Topic 5) being particularly dominant.

These results reveal distinct thematic patterns across the analyzed narratives, indicating that Chinese adolescents' EBSA narratives can be organized into seven thematically distinct clusters, some of which correspond to patterns reported in prior EBSA research, while others reflect systemic and institutional barriers (4, 7).

Taken together, these results demonstrate that EBSA among Chinese adolescents is structured around seven thematically distinct yet interrelated domains. This not only corroborates prior findings from Western contexts—such as the centrality of anxiety (1) and the protective role of supportive teacher–student relationships (2)—but also foregrounds systemic and institutional barriers (4) that have been less emphasized in earlier studies.

### 3.3 Validation results

The NVivo-based thematic analysis generated seven higher-order themes, corresponding closely to the seven clusters identified by BERTopic. As summarized in Table 1, the manual coding confirmed the prominence of anxiety-driven symptoms, family conflict and pressure, teacher–student relations, peer exclusion, post-COVID adjustment challenges, systemic barriers, and diminished belonging and agency. Subthemes and representative codes closely mirrored those extracted computationally. For instance, “my heart races before class” and “I panic when thinking of homework” were categorized under Anxiety-driven symptoms, echoing Topic 0 in the BERTopic model. Similarly, parental strictness (“Dad is too strict”) and divorce-related stress (“Home feels unstable”) were captured under Family conflict and pressure, consistent with Topic 2.

**Table 1:** Validation of BERTopic-derived themes through NVivo manual coding (*N* = 6,000 narratives).

Theme	Subtheme	Representative Codes
Anxiety-driven symptoms	Physiological responses	“My heart races before class”; “I feel dizzy at school”
	Emotional overwhelm	“I cry every morning”; “I panic when thinking of homework”
Family conflict & pressure	Parental strictness	“Dad is too strict”; “Mom sets unrealistic rules”
	Divorce & instability	“Parents divorced last year”; “Home feels unstable”
Teacher–student relations	Lack of support	“Teacher doesn't listen”; “I feel ignored in class”
	Harsh discipline	“Scolded in front of classmates”; “Teacher humiliates me”
Peer exclusion	Bullying/rumors	“Rumors about me”; “Friends isolate me at lunch”
Post-COVID difficulties	Workload shock	“Too much homework after reopening”; “I cannot catch up”
	Health-related anxiety	“Afraid of getting sick again”; “COVID made my anxiety worse”
Systemic barriers	Punitive policies	“School threatened fines”; “Parents blamed by school”
	Lack of resources	“No counselor available”; “Services not funded”
Loss of belonging & agency	Social isolation	“I feel like an outsider”; “Nobody cares if I attend”
	Powerlessness	“I have no say in class decisions”; “I just gave up”

Specifically, all seven BERTopic-derived themes showed direct correspondence with NVivo higher-order themes at the thematic level. Observed differences were confined to the level of thematic granularity rather than thematic presence. While the BERTopic model integrated teacher-related disciplinary experiences within broader relational difficulty clusters, NVivo coding further differentiated harsh disciplinary practices as a distinct subtheme under Teacher–student relations. Overall, substantial thematic overlap was observed between the BERTopic-derived themes and the manually coded NVivo themes, with minor differences reflecting differences in analytical resolution across methods.

## 4 Discussion

This study contributes to the expanding literature on EBSA by addressing three critical areas of innovation: research context, methodological approach, and substantive findings.

### 4.1 Cross-contextual validation through a novel research setting

A central contribution lies in the cross-context validation of EBSA constructs within a non-Western context. While the majority of EBSA studies to date have focused on Western primary and secondary school populations (1–3), the present study systematically examined the phenomenon among Chinese adolescents, drawing on 60,000 counseling-based narratives. By situating EBSA in China's unique socio-educational environment—characterized by high academic pressure, evolving family structures, and post-pandemic systemic challenges—this research provides empirical evidence on whether core mechanisms such as anxiety, family conflict, and teacher–student relationships are universal or contextually specific. The findings suggest substantial convergence with Western literature in highlighting anxiety and relational dynamics, while also identifying systemic and institutional barriers (e.g., inconsistent school–parent communication and punitive policies) as particularly salient in the Chinese context. This demonstrates the necessity of cross-cultural research for building a globally valid framework of EBSA.

Importantly, these convergences do not imply contextual equivalence. Rather, they indicate that similar psychological vulnerabilities are expressed and managed through distinct institutional arrangements in non-Western educational systems.

### 4.2 Methodological innovation with computational text analysis

Equally important, the study advances EBSA research by employing BERTopic combined with semantic network analysis to examine large-scale qualitative data. Traditional EBSA studies often rely on small sample interviews or surveys, limiting both generalizability and analytical depth (7). By contrast, this study applied transformer-based topic modeling to tens of thousands of adolescent narratives, enabling the detection of fine-grained semantic structures beyond simple frequency counts. Integrating co-occurrence heatmaps, semantic network analysis, and BERTopic modeling. This computational approach demonstrates how natural language processing can extend the reach of qualitative inquiry, making it possible to preserve cultural nuance while achieving scale and methodological rigor.

### 4.3 Substantive innovation in EBSA findings

The analysis uncovered seven distinct thematic domains—anxiety-driven physiological symptoms, sensory overload, family conflict, peer exclusion and teacher conflict, post-COVID adjustment difficulties, systemic barriers, and loss of belonging and agency. This typology makes several new contributions to EBSA research. First, it validates the cross-context relevance of established predictors such as anxiety (1) and relational support (2). Second, it foregrounds systemic and institutional barriers (4), which have received limited attention in prior Western-centered studies. Third, it highlights how pandemic disruptions have left lasting effects on adolescents' school engagement, consistent with but extending beyond recent post-COVID analyses (5). Together, these results broaden the conceptualization of EBSA by integrating individual, familial, school-based, and systemic levels within a culturally grounded thematic map (34).

By integrating manual NVivo coding with computational topic modeling, this study demonstrated methodological triangulation. The substantial convergence across both approaches reinforces the robustness of the thematic structures identified, while minor discrepancies provide additional nuance. This validation step addresses concerns about model-specific bias and strengthens confidence in the cross-contextual relevance of the findings.

Unlike much of the Western EBSA literature, which emphasizes therapeutic access and individualized support, the present findings foreground punitive governance practices, administrative surveillance, and asymmetric power relations between schools and families as salient features shaping EBSA experiences in the Chinese context.

### 4.4 Limitations and future directions

Despite its contributions, this study has several limitations. First, although the corpus of 60,000 narratives is unprecedented in scale, it was derived from a single national context. While the inclusion of NVivo-based manual thematic coding provided an important cross-method validation of the BERTopic results, the dataset itself remains limited to China. Future studies should incorporate multi-site or cross-cultural corpora to determine whether the thematic structures identified here are globally robust or shaped by local socio-educational systems.

Second, although the triangulation of computational and manual methods strengthened the robustness of the findings, both approaches relied on the same textual dataset. Future research could therefore extend validation by testing the thematic structure against independent corpora from other digital platforms (e.g., Weibo, Zhihu) or complementary data sources (e.g., classroom observations, parental interviews). Such cross-platform and multi-source validation would provide stronger evidence that the themes identified are not artifacts of a single dataset but represent stable dimensions of EBSA across contexts.

Third, although publicly available Xiaohongshu (RED) narratives provided valuable access to adolescents' spontaneous expressions of school distress, important limitations should be acknowledged. Student status on social media cannot be independently verified, and inclusion relied on explicit self-referential indicators (e.g., references to school schedules, homework, teachers, or examinations). Even among verified student-like posts, these narratives likely represent a selective subgroup of adolescents who are willing and able to articulate their experiences publicly online.

Consequently, the voices captured from RED may underrepresent students who are more withdrawn, digitally excluded, or reluctant to disclose distress in public or semi-public spaces. This introduces a potential visibility bias, whereby certain forms of EBSA experiences remain unheard. While the inclusion of institutional counseling transcripts partially mitigates this limitation, future research should integrate additional data sources—such as school-based interviews, parental reports, or longitudinal surveys—to more comprehensively capture the full spectrum of EBSA experiences.

Finally, while this study emphasizes thematic structures, it cannot establish causal pathways among identified factors. Longitudinal and intervention-based designs are needed to trace developmental trajectories and evaluate the effectiveness of targeted supports.

## 5 Conclusion

This study provides the first large-scale, computationally driven examination of EBSA among Chinese adolescents, offering both cross-context validation and culturally specific insights. By identifying seven thematic domains—spanning anxiety, sensory overload, family conflict, peer and teacher relations, post-COVID adjustment, systemic barriers, and loss of belonging—the findings highlight that EBSA is not only an individual or familial issue but also embedded within broader institutional and policy environments.

For Chinese schools, these results suggest the urgent need to strengthen multi-level support systems and to reconsider prevailing attendance governance practices. First, at the classroom level, fostering trust-based teacher–student relationships and creating emotionally safe learning environments can reduce the sense of isolation and anxiety that drives avoidance. Second, at the family–school interface, more collaborative communication channels should be established to replace punitive and blame-oriented approaches, ensuring that parents are engaged as partners rather than positioned as targets of administrative pressure or sanctions. Third, at the systemic level, schools and local education authorities should move toward integrated, cross-sector frameworks that coordinate psychological services, social support, and educational planning, particularly in addressing the long-term institutional consequences of COVID-19 disruptions.

In sum, the study underscores that reducing EBSA in China requires a shift from reactive, punitive responses to preventive, relational, and systemic strategies. By embedding emotional well-being into the core of educational practice, Chinese schools can not only mitigate school avoidance but also promote a culture of belonging and resilience that benefits all students.

## Data Availability

The original contributions presented in the study are included in the article/Supplementary Material, further inquiries can be directed to the corresponding author.
